# Vasorelaxant and Antioxidant Activities of *Spilanthes acmella* Murr.

**DOI:** 10.3390/ijms9122724

**Published:** 2008-12-18

**Authors:** Orapin Wongsawatkul, Supaluk Prachayasittikul, Chartchalerm Isarankura-Na-Ayudhya, Jutamaad Satayavivad, Somsak Ruchirawat, Virapong Prachayasittikul

**Affiliations:** 1 Department of Pharmacology, Faculty of Medicine, Srinakharinwirot University, Bangkok 10110, Thailand. E-Mail: orapinw@swu.ac.th; 2 Department of Chemistry, Faculty of Science, Srinakharinwirot University, Bangkok 10110, Thailand; 3 Department of Clinical Microbiology, Faculty of Medical Technology, Mahidol University, Bangkok 10700, Thailand. E-Mail: mtcis@mahidol.ac.th; 4 Laboratory of Pharmacology, Chulabhorn Research Institute, Bangkok 10210, Thailand. E-Mail: jutamaad@cri.or.th; 5 Laboratory of Medicinal Chemistry, Chulabhorn Research Institute, Bangkok 10210, Thailand. E-Mail: somsak@cri.or.th

**Keywords:** *Spilanthes acmella* Murr., vasorelaxant, antioxidant activities, nitric oxide, prostacyclin, thoracic aorta

## Abstract

This study reports the effect of *Spilanthes acmella* Murr. extracts on phenylephrine-induced contraction of rat thoracic aorta as well as their antioxidant activity. Results show that the extracts exert maximal vasorelaxations in a dose-dependent manner, but their effects are less than acetylcholine-induced nitric oxide (NO) vasorelaxation. Significant reduction of vasorelaxations is observed in both *N*^G^-nitro-l-arginine methyl ester (l-NAME) and indomethacin (INDO). In the presence of l-NAME plus INDO, synergistic effects are observed, leading to loss of vasorelaxation of both acetylcholine and the extracts. Similarly, the vasorelaxations of the extracts are completely abolished upon the removal of endothelial cells. This demonstrates that the extracts exhibit vasorelaxation *via* partially endothelium-induced NO and prostacyclin in a dose-dependent manner. Significantly, the ethyl acetate extract exerts immediate vasorelaxation (ED_50_ 76.1 ng/mL) and is the most potent antioxidant (DPPH assay). The chloroform extract shows the highest vasorelaxation and antioxidation (SOD assay). These reveal a potential source of vasodilators and antioxidants.

## 1. Introduction

*Spilanthes acmella* Murr. (para-cress or toothache plant) is a medicinal plant of the Compositae family [1]. It is known in Thai as Phak-Kratt Huawaen and has long been used as a traditional medicine for toothache, headache and treatment of asthma, rheumatism, fever, sore throat and haemorrhoids [1–3]. Its root decoction has been used as a laxative and diuretic drug [2, 4]. It is an annual or short-lived herb, spreads in low open places and requires moist soil [5]. Its flowers and leaves have a pungent taste, accompanied with tingling and numbness [2].

Constituents found in *Spilanthes acmella* Murr. were spilanthol, isobutylamide derivatives [6, 7], *α*-and *β*-amyrin esters, stigmasterol, myricyl alcohol including sitosterol glucosides [8] and triterpenoidal saponins [9]. Spilanthol showed interesting bioactivities, e.g. strong local anesthetic [10], analgesic [11, 12] and insecticidal activities [3]. Crude flower head extracts of the plant exhibited potent ovicidal, marked larvicidal (LC_50_ of 61.43 ppm) and pupicidal activities [13, 14]. The extracts were also shown to exhibit pancreatic lipase inhibition which has potential as candidates for weight loss and obesity control [15]. In addition, the crude flower head extracts displayed strong diuretic action which caused marked increase in urine Na^+^, K^+^ and a reduction in the osmolarity of urine [16]. Moreover, it has been used as anti-toothache formulation for pain relieving within 2–10 min and also useful in treating the swelling and infection of gums [17]. The *Spilanthes acmella* is one of the active ingredients in compositions for acute- or long-term treatment of microbial infections, particularly, oral pathogenic microorganisms, dental caries, periodontosis, gum disease, gum bleeding and/or plaque reduction [18]. In addition, its root powder extract is very effective in treating HIV/AID infection [19]. Presently, cardiovascular disease is one of the most important public health concerns, accountable for morbidity and mortality. Thus new vasoactive ingredients that influence the endothelial cells, which are key regulating cells in the vessel wall, are the striking areas. So far, the effect of *Spilanthes acmella* Murr. on vascular function has not been reported. In this regard, it was of great interest to explore its medicinal value with regards to cardiovascular functions such as regulation of vascular tone. In the present study, we have investigated the effects of *Spilanthes acmella* Murr. extracts on rat thoracic aorta and its mechanism of action as well as its antioxidant activity.

## 2. Results

### 2.1. Components and HPLC (high performance liquid chromatography) profiles of the extracts

Preliminary screening of components in the *Spilanthes acmella* Murr. extracts was performed using thin layer chromatography (TLC) chromatograms, IR and ^1^H-NMR spectra. It was found that hexane, chloroform, ethyl acetate and methanol extracts showed UV absorption on TLC. The ethyl acetate and methanol extracts exhibited very strong absorptions, compared to the chloroform extract. The IR spectra of the extracts showed absorptions (cm^−1^) corresponding to OH (3360–3385), CH (2850–2942), CO (1710–1736), 1454–1574 and C-O (1047–1385) functions. The ethyl acetate and methanol extracts exhibited very strong OH and CO (ester or acid) and strong C-O absorptions. The chloroform extract displayed strong OH, medium CO (ester or acid) and C-O absorptions. The hexane extract showed strong CO (ester or acid), but medium OH and weak C-O absorptions. All the extracts showed strong CH absorptions, the the hexane extract displaying the strongest ones. The ^1^H-NMR spectra of the hexane and chloroform extracts exhibited the presence of triterpenes (δ 0.9–2.4, 3.4–3.7, 4.1–4.3 and 5.3–5.4). The ethyl acetate extract showed the signals of triterpenes and aromatics (δ 0.84–2.4, 3.5–4.3, 5.3–5.5 and 6.8–7.8). The methanol extract displayed the presence of aromatic protons (δ 6.8–8.4).

Based on the IR and ^1^H-NMR data, it may be concluded that the hexane and chloroform extracts contain triterpenes and long chain hydrocarbon esters or alcohols. The ethyl acetate extract contains triterpene esters and phenolic compounds, while the components of the methanol extract are presumably phenolic compounds and esters.

**Figure 1:**
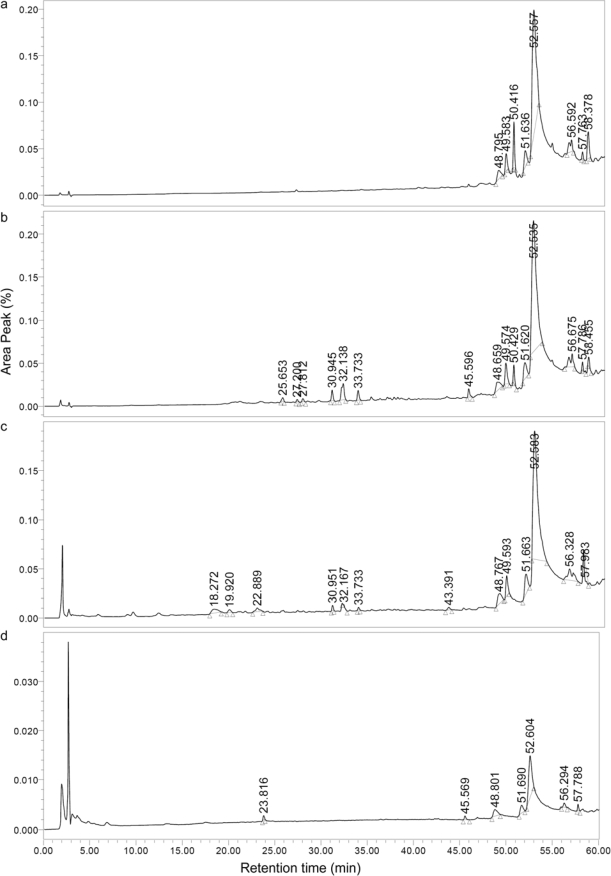
HPLC profiles of plant extracts in hexane (a, 228.0 nm), chloroform (b, 228.0 nm), ethyl acetate (c, 228.0 nm), and methanol (d, 280.0 nm).

Profiles of the extracts were obtained by reversed phase HPLC running from a more polar to less polar mobile phase (Figure 1). Major nonpolar components of the extracts appeared in the 48.80–58.38 min retention time (RT) range. In the hexane extract the major component (66.06%) was found at a RT of 52.56 min, while th major components of the chloroform (65.72%), ethyl acetate (67.34%) and methanol (64.18%) extracts were observed at RTs of 52.54, 52.58 and 52.60 min, respectively. It was noted that minor contents (0.22–4.72%) of polar components in chloroform and ethyl acetate extracts appeared at RTs of 18.27–33.73 min. However, the methanol extract showed a minor polar component (2.64%) at a RT of 23.82 min (Table 1).

### 2.2. Vasorelaxant activity

Effects of *Spilanthes acmella* Murr. extracts (hexane, chloroform, ethyl acetate and methanol) on vascular function of rat thoracic aorta precontracted with phenylephrine (PE) were investigated under various conditions; in the presence or absence of inhibitors, namely, *N*^G^-nitro-l-arginine methyl ester (l-NAME) and indomethacin (INDO) and under removal of functional endothelial cells. In addition, the effects of acetylcholine (ACh) as a positive control, sodium nitroprusside (SNP) a negative control and vehicle such as polyethyleneglycol (PEG) were studied. Results confirmed that the vasorelaxation of ACh was related to nitric oxide (NO).

### 2.3. Effect of Spilanthes acmella Murr. extracts on the vascular function of rat thoracic aorta in the absence or presence of a NOS inhibitor (L-NAME, 1 mM)

#### 2.3.1. Hexane extract

**Figure 2:**
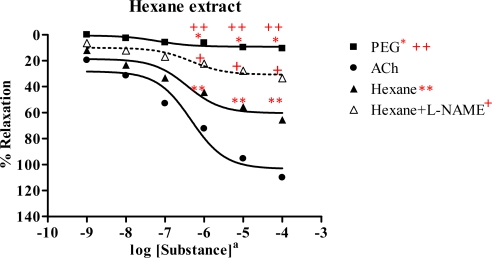
Effect of hexane extract on the vascular function of rat thoracic aorta in the absence or presence of NOS inhibitor (l-NAME, 1 mM) compared with those of ACh and PEG. Data represent as means ± s.e.m. of 6 experiments, each performed in duplicate. ★ *p* < 0.05, ACh versus PEG, ★★ *p* < 0.05, ACh versus hexane extract, + *p* < 0.05, hexane extract versus hexane extract +l-NAME, ++ *p* < 0.05, PEG versus hexane extract a: hexane extract as mg/mL, ACh as molar

In the rat aortic ring preparation, the hexane extract exerted vasorelaxation in the dose-dependent manner, but PEG showed no effect on the vessels (Figure 2 and Table 2). The activity of the hexane extract reached up to 65.7% of the R_max_ induced by ACh (110.1%) showing ED_50_ 3.60×10^−7^ mg/mL and 4.66×10^−7^ M, respectively. This indicated that the hexane extract likely acted as a partial agonist. In the presence of a NOS inhibitor (l-NAME), the dose-response curve of hexane extract was shifted to the right, with R_max_ 33.5% and ED_50_ 4.80×10^−7^ mg/mL. This suggested that the hexane extract caused vasorelaxation by partially producing NO from the endothelial cells, while PEG had no effect on induction of vasorelaxation.

#### 2.3.2. Chloroform extract

**Figure 3:**
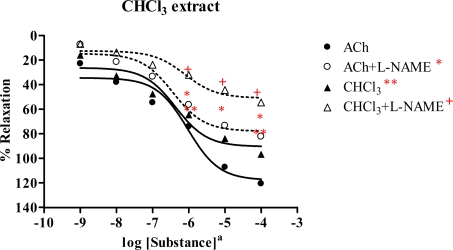
Effect of CHCl_3_ extract and ACh on the vascular function of rat thoracic aorta in the absence or presence of 1 mM l-NAME. Data represent as means ± s.e.m. of 5–6 experiments, each performed in duplicate. ★ *p* < 0.05, ACh versus ACh+l-NAME, ★ ★ *p* < 0.05, ACh versus CHCl_3_ extract, + *p* < 0.05, CHCl_3_ extract versus CHCl_3_ extract+l-NAME a: CHCl_3_ extract as mg/mL, ACh as molar

Similarly, the chloroform extract showed dose-dependent vasorelaxation (Figure 3 and Table 2). The R_max_ of ACh and the chloroform extract were 120.6 and 96.6%, with ED_50_ 9.43×10^−7^ M and 4.28×10^−7^mg/mL, respectively. In the presence of l-NAME, the dose-response curve of chloroform extract was shifted to the right. The extract showed R_max_ 54.4% and ED_50_ 1.00×10^−6^ mg/mL, whereas R_max_ and ED_50_ of ACh were 82.1% and 3.61×10^−7^ M, respectively. This demonstrated that the chloroform extract exhibited vasorelaxation by partly production of NO from the endothelial cells.

#### 2.3.3. Ethyl acetate extract

**Figure 4:**
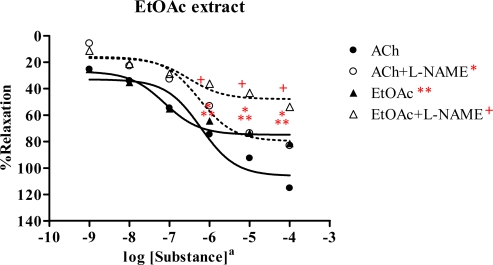
Effect of EtOAc extract and ACh on the vascular function of rat thoracic aorta in the absence or presence of 1 mM l-NAME. Data represent as means ± s.e.m. of 5 experiments, each performed in duplicate. ★ *p* < 0.05, ACh versus ACh+l-NAME, ★ ★ *p* < 0.05, ACh versus EtOAc extract, + *p* < 0.05, EtOAc extract versus EtOAc extract+l-NAME a: EtOAc extract as mg/mL, ACh as Molar

A similar result was obtained from ethyl acetate extract, showing vasorelaxation in the dose-dependent manner (Figure 4 and Table 2). The ACh and plant extract exerted R_max_ 115.5 and 81.6%, with ED_50_ of 5.91×10^−7^ M and 7.61×10^−8^ mg/mL, respectively. This showed that the ethyl acetate extract possibly acted as a partial agonist. In the presence of l-NAME, the dose-response curve of the extract was shifted to the right with R_max_ 54.0%, ED_50_ 3.88×10^−7^ mg/mL, whereas the R_max_ and ED_50_ of ACh were 83.4% and 5.65×10^−7^ M, respectively. This suggested that the ethyl acetate extract caused vasorelaxation by partly synthesis of NO from the endothelial cells.

#### 2.3.4. Methanol extract

**Figure 5:**
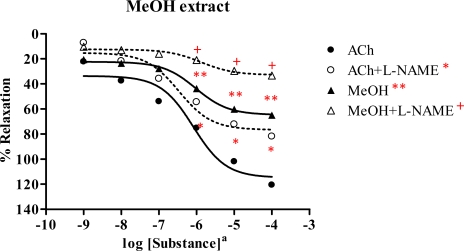
Effect of MeOH extract and ACh on the vascular function of rat thoracic aorta in the absence and presence of 1 mM L-NAME. Data represent as means ± s.e.m. of 6 experiments, each performed in duplicate. ★ *p* < 0.05, ACh versus ACh+l-NAME, ★ ★ *p* < 0.05, ACh versus MeOH extract, + *p* < 0.05, MeOH extract versus MeOH extract+l-NAME a: MeOH extract as mg/mL, ACh as molar

The methanol extract displayed vasorelaxant activity in a dose-dependent manner (Figure 5 and Table 2). The R_max_ of ACh and extract were 120.7 and 65.1% with ED_50_ 8.18×10^−7^ M and 9.55×10^−7^ mg/mL, respectively. This indicated that the extract possibly acted as partial agonist. In the presence of l-NAME, the dose-response curve of extract was shifted to the right with R_max_ 33.4% and ED_50_ 1.38×10^−6^ mg/mL, whereas the R_max_ and ED_50_ of ACh were 82.0% and 3.43×10^−7^M, respectively. This illustrated that the methanol extract exerted vasorelaxation by partial NO production from the endothelial cells.

### 2.4. Effect of Spilanthes acmella Murr. on the vascular function of rat thoracic aorta in the absence of endothelial cells

**Figure 6:**
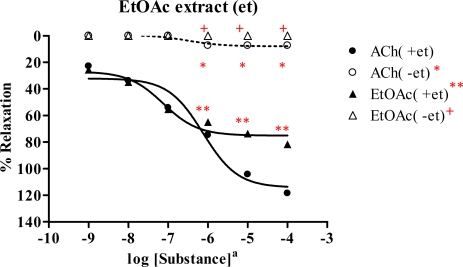
Effect of EtOAc extract and ACh on the vascular function of rat thoracic aorta under removal of endothelium (−et) compared with intact endothelium (+et). Data represent as means ± s.e.m. of 4 experiments, each performed in duplicate. ★ *p* < 0.05, ACh(+et) versus ACh(−et), ★★ *p* < 0.05, ACh(+et) versus EtOAc extract (+et), + *p* < 0.05, EtOAc extract (+et) versus EtOAc extract (−et) a: EtOAc extract as mg/mL, ACh as Molar

The vasorelaxation of *Spilanthes acmella* Murr. extracts was examined with and without intact endothelial cells, comparing with ACh. The results (Table 3) illustrated that the vasorelaxation of all the tested extracts was abolished when endothelial cells were removed. An example, the dose-response curve of the ethyl acetate extract is shown in Figure 6. Similar results were observed for the control, ACh. This confirmed that the vasorelaxant activity of *Spilanthes acmella* Murr. extracts was mediated *via* endothelial cells producing NO.

### 2.5. Effect of Spilanthes acmella Murr. on the vascular function of rat thoracic aorta in the presence of a cyclooxygenase inhibitor (INDO)

**Figure 7:**
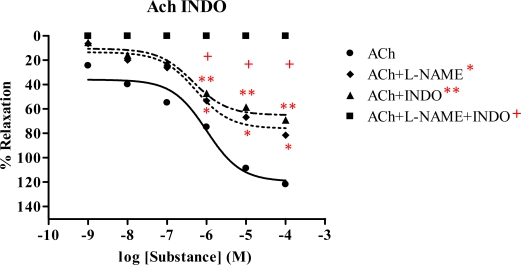
Effect of ACh in the presence of l-NAME plus INDO on the vascular function of rat thoracic aorta precontracted with PE. Data represent as means ± s.e.m. of 5 experiments, each performed in duplicate. ★ *p* < 0.05, ACh versus ACh+l-NAME, ★★ *p* < 0.05, ACh versus ACh+INDO, + *p* < 0.05, ACh versus ACh+l-NAME+INDO

**Figure 8:**
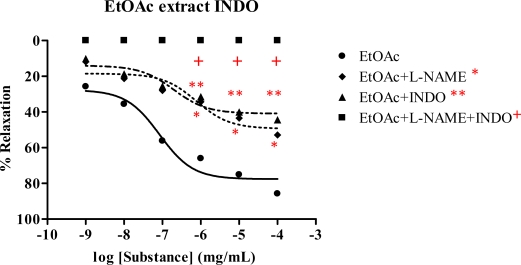
Effect of EtOAc extract on the vascular function of rat thoracic aorta in the presence of INDO compared with l-NAME and with l-NAME plus INDO. Data represent as means ± s.e.m. of 5 experiments, each performed in duplicate. ★ *p* < 0.05, EtOAc extract versus EtOAc extract+l-NAME, ★★*p* < 0.05, EtOAc extract versus EtOAc extract+INDO, + *p* < 0.05, EtOAc extract versus EtOAc extract+l-NAME+INDO

**Figure 9:**
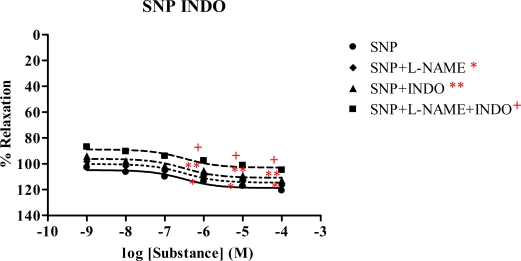
Effect of SNP in the presence of l-NAME, INDO and l-NAME plus INDO on the vascular function of rat thoracic aorta. Data represent as means ± s.e.m. of 6 experiments, each performed in duplicate. ★★ *p* < 0.05, SNP versus SNP+l-NAME, ★★ *p* < 0.05, SNP versus SNP+INDO, + *p* < 0.05, SNP versus SNP+l-NAME+INDO

The vasorelaxation of *Spilanthes acmella* Murr. extracts was studied in the presence of INDO (1 mM), comparing with l-NAME (1 mM) and l-NAME plus INDO. The results are summarized in Table 4 and some dose-response curves are shown in Figures 7–9. It was observed that in each experiment with l-NAME (1 mM) or INDO (1 mM) the vasorelaxation was reduced in a dose-dependent manner. The inhibition effect of the INDO was stronger than that of l-NAME. However, the antagonist effects of l-NAME or INDO were more pronounced in the case of l-NAME plus INDO. Such effects were not observed in the SNP dose-response curves. Interestingly, the vasorelaxations were abolished by all the tested extracts and by the ACh in the presence of l-NAME (1 mM) plus INDO (1 mM), but no significant change was noted in the case of SNP. The results confirmed that the extracts caused partial vasorelaxation through endothelial cells producing NO and prostacyclin (PGI_2_).

### 2.6. Antioxidant activity

**Figure 10:**
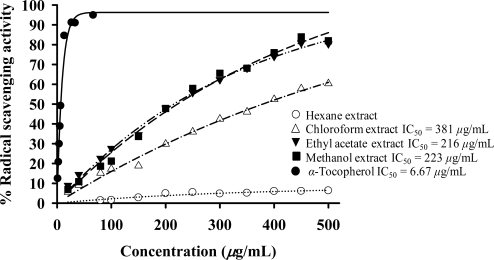
Radical scavenging activity of *α*-tocopherol and extracts.

The antioxidant activity of *Spilanthes acmella* Murr. extracts was measured using DPPH and SOD assays. The results (Table 5) showed that all the tested extracts exhibited antioxidative activity. In DPPH assay (Figure 10) at 200 *μ*g/mL, the ethyl acetate and methanol extracts displayed comparable activity and the highest radical scavenging activity (47.90 and 47.76%) with IC_50_ 216 and 223 *μ*g/mL, while *α*-tocopherol (a positive control) showed antioxidant activity with IC_50_ 6.67 *μ*g/mL. The chloroform extract exhibited 29.82% radical scavenging activity. The hexane extract produced some activity (4.90% radical scavenging).

**Figure 11:**
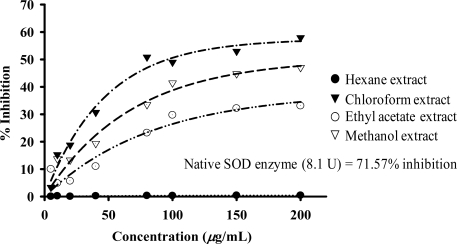
Superoxide dismutase activity of crude extracts.

The SOD assay at 200 *μ*g/mL (Figure 11), the chloroform extract showed the highest antioxidant activity (57.92% NBT inhibition). The ethyl acetate and methanol extracts exhibited weak to moderate SOD activity.

## 3. Discussion

We have disclosed the effect of *Spilanthes acmella* Murr.extracts on phenylephrine-induced contraction of rat thoracic aorta. All the tested plant extracts elicit maximal vasorelaxations in a dose-related manner, although such vasorelaxations are less than those produced by ACh. The vasorelaxations are exerted by partial production of NO from functional endothelial cells, which is demonstrated by a significant reduction of the activity in the presence l-NAME (Table 2).

The chloroform extract exhibited the highest R_max_ of 96.6%, compared to the other extracts. The ethyl acetate extract shows strong vasorelaxation (R_max_ 81.6%), while the nonpolar hexane and polar methanol extracts exert comparable activity, with R_max_ 65.67 and 65.09%, respectively. Significantly, the ethyl acetate extract exhibits immediate vasorelaxation with ED_50_ 7.61×10^−8^ mg/mL (Figure 4) when compared to the chloroform extract showing ED_50_ 4.28×10^−7^ mg/mL (Figure 3). This perhaps due to the fact that the ethyl acetate extract has higher affinity for the receptor than the chloroform extract and thus the ethyl acetate extract is the most potent vasorelaxant. Furthermore, the vasorelaxant activities of the tested extracts were all abolished by the removal of functional endothelial cells (Table 3 and Figure 6). This again confirms that the vasorelaxation of the extracts is modulated via NO production by endothelial cells, a fact which is known for Ach involvement in mediating vasorelaxation by NO, PGI_2_ and endothelium-derived hyperpolarizing factor [20–22]. Therefore, a set of experiments was studied in the presence of INDO (1 mM) compared with l-NAME (1mM). The results (Table 4) show that the vasorelaxation of the tested extracts (Figure 8) and ACh (Figure 7) is significantly reduced in a dose-dependent manner when compared to that of in the presence of l-NAME. Particularly, the INDO exhibits stronger reduction of such vasorelaxation than the l-NAME, but none was not observed for the SNP (Figure 9). Additionally, in the presence of INDO (1 mM) plus l-NAME (1 mM), synergistic effects are observed, leading to complete loss of the activity of the tested extracts and ACh. However, such an effect is not markedly noticeable for the SNP. These data confirm that the vasorelaxant activity of *Spilanthes acmella* Murr. is exerted by functional endothelial cells producing partial synthesis of NO and PGI_2_ which are inhibited by l-NAME and INDO, respectively. NO is an important signaling molecule implicated in cardiovascular functions such as vascular tone. PGI_2_, endothelium-derived relaxing factor (EDRF), is a powerful vasorelaxant and antioxidant. It exerts antifibrotic properties preventing the development of fibrosis and cirrhosis in liver diseases including antiplatelet aggregation. PGI_2_ is clinically used for treatment of pulmonary hypertension and portopulmonary hypertension [23].

Many studies have reported the role of NO related to superoxide radical (O_2_^.−^) [24]. Thus, the antioxidative activity of the extracts was evaluated. It was found that the plant extracts exhibited antioxidant activity at 200 *μ*g/mL. In a DPPH assay, the polar ethyl acetate and methanol extracts exerted the highest radical scavenging activity, while the chloroform extract shows the highest SOD activity. The nonpolar hexane extract produces some activity in both the DPPH and SOD assays.

It is noted that the ethyl acetate extract is the most potent, with immediate vasorelaxation and with the highest radical scavenging activity. This is presumably due to the presence of bioactive polar phenolic and triterpenoid ester compounds in the extract. Moreover, the chloroform extract possesses the highest R_max_ accompanied by the highest SOD activity. This may result from the presence of triterpenoids and fatty alcohols or esters in this extract. Moderate vasorelaxations are observed for the nonpolar hexane and polar methanol extracts. This suggests that both hydrophobic and hydrophilic constituents show comparable vasorelaxant activity (Table 2). At this point, the ethyl acetate extract is the most promising vasorelaxant, with a significant ED_50_ of 76.1 ng/mL and it is also the most active antioxidant (DPPH assay). It has been reported that flavonoid, phenolic, polyphenol and triterpenoid compounds exhibit both vasorelaxant and antioxidant activities, in some cases behaving as either vasorelaxants or antioxidants [24–26]. Therefore, our results imply a good correlation between vasorelaxant and antioxidant activities deriving from a diverse group of compounds.

It is well recognized that endothelium NO is diminished by reacting with O_2_^.−^ to form peroxynitrite as a potent oxidant, which is inhibited by antioxidant, thus in turn improving NO induced vasorelaxation [24, 27–29]. This was evidenced by phenolic antioxidants such as prinsepiol isolated from roots of *Valeriana prionophylla* [24]. A number of flavonoids exerted vasorelaxation through endothelium produced NO, e.g. chrysin (5, 7-dihydroxyflavone) presented in honey, propolis, fruits, vegetables and beverages [30–32], isoliquiritigenin isolated from various plants such as *Dahlia variabilis* [33] and recently from *Hydnophytum formicarum* Jack. [34]. Triterpenoids are commonly found in plant species, for instances, oleanolic acid and erythrodiol elicit vasorelaxation *via* endothelium induced NO [26]. In addition, root extracts of *Caesalpinia benthamiana*, which are rich in phenolic compounds, e.g. gallic acid, resveratrol and tannin, had significant vasorelaxant and powerful scavenging activity versus O_2_.^−^ [25]. Some plant extracts of *Casimiroa edulis* and *Mammea africana* (Guttiferae) caused vasorelaxation through endothelium dependent producing NO [35, 36]. The vasorelaxation was also mediated by both endothelium dependent and endothelium independent pathways [37]. Moreover, extracts of ethnomedical plant used as antihypertensive and anti-stroke exhibited their vasorelaxants *via* endothelium dependent producing NO or *via* direct stimulation of NO release. Examples are *Diospyros kaki* Thunb., *Polygonum aviculare* L., *Magnolia liliflora* Desr., *Sorbus commixta* Hedl, *Selaginella tamariscina* Spr. and *Guazuma ulmifolia* [38, 39].

In this study, *Spilanthes acmella* Murr. extracts produce vasorelaxation *via* partial release of NO and PGI_2_ from endothelium as well as showing antioxidant activities. Some countries in Asia including Thailand use this plant as fresh vegetable and cooking ingredient [40]. This demonstrates beneficial effects and applications of the *Spilanthes acmella* Murr. to medical uses and as a health food.

## 4. Experimental Section

### 4.1. Materials

#### 4.1.1. Chemicals

L-Phenylephrine hydrochloride, sodium nitroprusside, *N*^G^-nitro-L-arginine methyl ester, acetylcholine, ketamine hydrochloride and indomethacin were obtained from Sigma Chemical Co. (St. Louis, MO, USA). Polyethyleneglycol was purchased from Fluka. Plant extracts were dissolved in PEG, while methanol extract was dissolved in 0.9% normal saline. Then the solutions were further diluted by normal saline.

#### 4.1.2. Plant materials

Aerial parts of *Spilanthes acmella* Murr. were collected from Nakornsrithammarat Province, Thailand. It has been identified (BKF 112361) by The Forest Herbarium, Royal Forestry Department. A voucher specimen has been deposited at Department of Chemistry, Faculty of Science, Srinakharinwirot University, Thailand.

### 4.2. Methods

#### 4.2.1. Extraction

The air dried *Spilanthes acmella* Murr. (1,050 g) was ground and extracted with hexane (3×5 days), followed by filtration. The filtrates were combined and evaporated *in vacuo* to give a crude hexane extract (11 g). Similarly, the extraction was performed using chloroform, ethyl acetate and methanol to obtain the corresponding chloroform (10 g), ethyl acetate (18 g) and methanol (31 g) extracts, respectively.

#### 4.2.2. Components and HPLC profiles of the extracts

TLC chromatograms of the extracts were run on pre-coated silica gel 60F_254_ (Merck). Phenolic groups were detected by UV fluorescence. Their IR (neat, Perkin Elmer Spectrum One FT–IR spectrometer) and ^1^H-NMR (CDCl_3_ or DMSO-d_6_, Bruker Avance 300 MHz) spectra were recorded. HPLC profiles were performed with flow rate 1 mL/min using a Hichrom Exsil 100-5ODS column (25 cm length with 4.6 mm diameter) and a Waters 2996 Photodiode Array Detector. The tested extracts were prepared by dissolving 1 mg in 1 mL methanol. Aliquots of 20 *μ*L were injected using gradient elution with decreasing polarity from H_2_O: CH_3_OH (9:1) to CH_3_OH and running for 100 min. Peaks of components were detected at 228.0 nm (hexane, chloroform and ethyl acetate extracts) and at 280.0 nm (methanol extract).

#### 4.2.3. Biological activity

The study was performed using the hexane, chloroform, ethyl acetate and methanol extracts of *Spilanthes acmella* Murr., obtained as described above.

##### 4.2.3.1. Vasorelaxant assay

###### 4.2.3.1.1. Isometric tension measurements

The protocols for handling animals were approved by the Animal Care Committee at the Srinakharinwirot University and done at the National Laboratory Animal Centre, Mahidol University. Male Sprague-Dawley rats (170–250 g) were anesthetized with intraperitoneal ketamine hydrochloride (0.05 mL/kg). The thoracic aorta was quickly removed to cold Kreb-Henseleit buffer containing (mM): 118 NaCl; 4.7 KCl; 1.2 KH_2_PO_4_; 1.2 MgSO_4_·7H_2_O; 11.0(+)-glucose; 25.0 NaHCO_3_; and 2.5 CaCl_2_·2H_2_O, pH 7.4, aerated with 95% O_2_, 5% CO_2_. After removed debris tissue, the vessel was cut into rings, each 2–3 mm-long and hanged in the organ bath containing Kreb-Henseleit solution at 37 °C, aerated with 95% O_2_, 5% CO_2_ and also connected to a force-displacement transducer (Model MLTO50 Force transducer Range: 50, P.R. China) and equilibrated for 50–60 min under a 1 g resting tension. During an incubation period, the Kreb-Henseleit solution was changed every 20 min. After the incubation period, the maximal contraction of the rings was determined with high dose of PE (10^−5^ M) and then washed 5 times until resting tension was recovered. Isometric tension [41] was recorded by Macintosh MacLab 4E AD Instrument connected to computer hard drive. The endothelial intact was examined using high dose of ACh (10^−5^ M) at a level of submaximal tension. If the relaxation response to ACh was less than 80%, the ring would be discarded. Then, the ring was washed 5 times to remove the residue of ACh. The vessel was again equilibrated for 50–60 min and the responses of vessel were performed by the following protocols. Submaximal contraction was induced using PE (10^−7^ M), then cumulative dose-response curves to the agonists (10^−9^–10^−4^ M or mg/mL). Finally, the dose-response curve of SNP was performed in order to test the functional vessel. With inhibitors (L-NAME or INDO) or vehicle, the vessels were pretreated with such compounds prior to submaximal contraction with PE then examine the endothelial response to the tested compounds. After each cumulative dose-response curve, the thoracic aorta preparation was washed and equilibrated 50–60 min before working on the next dose-response curve of tested compounds.

###### 4.2.3.1.2. Statistical analyses

The unpaired two-tailed Student’s t test and one-way ANOVA were used in the statistical analysis when appropriate. *Post-hoc* comparisons of individual groups were performed using the Tukey-Kramer test. The ED_50_ values for the vasorelaxants were calculated using nonlinear regression analyses (GraphPad Prism 4, GraphPad Software Inc., Sandiego, CA, USA). A *p*-value less than 0.05 was considered significant. The data were expressed as mean ± s.e.m. for the number of animals.

##### 4.2.3.2. Antioxidative assay

Two assay methods were used: 2,2-diphenyl-1-picrylhydrazyl (DPPH) and superoxide dismutase (SOD). The antioxidative activity of the crude extracts was elucidated by the DPPH radical scavenging assay [42]. When DPPH (a stable purple color) reacts with an antioxidant, it is reduced to yield a light-yellow colored diphenylpicrylhydrazine. Color changes can be spectrophotometrically measured. In this study, experiment was initiated by preparing 0.2 mM solution of DPPH in methanol. One mL of this solution was added sample solution (1 mg/mL dissolved in methanol, 0.5 mL). After 30 min, absorbance was measured at 517 nm and the percentage of radical scavenging activity was calculated from the following equation:

% Radical scavenging = (1-Abs.sample/Abs.cont)×100

where Abs.cont is the absorbance of the control reaction and Abs.sample is the absorbance in the presence of sample.

The SOD activity was assayed by measuring inhibition of the photoreduction of nitro blue tetrazolium (NBT) [43]. The indirect assay is comprised of several reactions: the photochemically excited riboflavin was first reduced by methionine into a semiquinone, which donated an electron to oxygen to form the superoxide source. The superoxide readily converted NBT into a purple formazan product. In this regard, the SOD activity was inversely related to the amount of formazan formation.

## 5. Conclusions

Our findings report for the first time that *Spilanthes acmella* Murr. extracts possess vasorelaxant and antioxidant activities. The plant extracts elicit vasorelaxations *via* partially endothelium induced NO and PGI_2_ in a dose-dependent manner. However, other underlying mechanisms may participate. Significantly, the ethyl acetate extract exhibits immediate vasorelaxation in nanogram levels and is the most potent antioxidant in the DPPH assay. The chloroform extract displays the highest vasorelaxation with the highest antioxidant (SOD assay). Furthermore, the nonpolar hexane and polar methanol extracts show moderate vasorelaxant activity. This demonstrates an important role for *Spilanthes acmella* Murr. as a new natural source of vasodilators and antioxidants. Furthermore, the results provide a guideline to further isolate bioactive ingredient, essentially, from chloroform and ethyl acetate extracts. However, the methanol and hexane extracts are also interesting to explore. Hopefully, new constituents will be isolated and investigated for bioactivities. Particularly, mechanism of vasorelaxation in more details involved EDHF *via* different potassium channels will be investigated.

## Figures and Tables

**Table 1:** Retention times and area peaks of plant extracts.

Extract	RT (min)	Area (%)	Extract	RT (min)	Area (%)
Hexane	48.80	3.86	Chloroform^b^	25.65	0.61
	49.58	4.20		27.20	0.37
	50.42	7.79		27.81	0.55
	51.64	5.38		30.95	1.69
	52.56	66.06		32.14	4.72
	56.59	5.09			
	57.76	0.97	Ethyl acetate^b^	18.27	2.55
	58.38	6.64		19.92	0.84
				22.89	1.57
Chloroform^a^	52.54	65.72		30.95	0.72
				32.17	0.22
Ethyl acetate^a^	52.58	67.34		33.73	0.38
Methanol^a^	52.60	64.18	Methanol^b^	23.82	2.64

^a^ Minor contents at RT range 48–58 min are similar to hexane extract, but data are not shown.

^b^ Minor contents of polar components at RT range 18.27–33.73 min.

**Table 2:** Vasorelaxant activity of *Spilanthes acmella* Murr. extracts on rat thoracic aorta.

Compound	Vasorelaxant activity			
	Without l-NAME		With l-NAME (1 mM)	
	R_max_ (%)	ED_50_ (mg/mL)	R_max_ (%)	ED_50_ (mg/mL)
Hexane extract^a^	65.67 ± 0.984	3.601×10^−7^	33.49 ± 1.122	4.805×10^−7^
ACh^a^	110.08 ± 1.801	4.657×10^−7^	NA	NA
Chloroform extract^b^	96.64 ± 1.112	4.279×10^−7^	54.38 ± 0.575	1.003×10^−6^
ACh^a^	120.63 ± 1.671	9.428×10^−7^	82.15 ± 1.158	3.611×10^−7^
Ethyl acetate extract^b^	81.64 ± 0.530	7.614×10^−8^	53.99 ± 1.517	3.885×10^−7^
ACh^b^	115.47 ± 0.951	5.912×10^−7^	83.36 ± 1.169	5.650×10^−7^
Methanol extract^a^	65.09 ± 0.409	9.550×10^−7^	33.38 ± 0.403	1.375×10^−6^
ACh^a^	120.69 ± 0.220	8.183×10^−7^	82.01 ± 0.582	3.427×10^−7^
ACh^b^	119.20 ± 0.344	7.878×10^−7^	80.86 ± 0.368	4.163×10^−7^
SNP^b^	120.53 ± 2.270	1.727×10^−7^	114.84 ± 0.716	3.171×10^−7^

a: data obtained from 6 experiments

b: data obtained from 5 experiments ED_50_ of ACh and SNP are expressed as molar

NA: not tested, *p* < 0.05

**Table 3:** Effect of endothelial cells on vasorelaxant activity of *Spilanthes acmella* Murr. extracts.

Compound^a^	Vasorelaxant activity			
	+Et		−Et	
	R_max_ (%)	ED_50_ (mg/mL)	R_max_ (%)	ED_50_ (mg/mL)
Hexane extract	64.82 ± 0.870	2.556×10^−7^	0	-
ACh	104.31 ± 1.595	2.440×10^−7^	7.16 ± 2.010	3.128×10^−7^
Chloroform extract	98.00 ± 0.694	4.422×10^−7^	5.64 ± 0.080	3.128×10^−7^
ACh	120.36 ± 0.823	7.534×10^−7^	7.42 ± 0.159	3.128×10^−7^
Ethyl acetate extract	81.68 ± 0.682	7.638×10^−8^	0	-
ACh	118.58 ± 0.550	7.477×10^−7^	7.42 ± 0.159	3.128×10^−7^
Methanol extract	65.62 ± 0.651	1.002×10^−6^	0	-
ACh	119.93 ± 0.495	7.967×10^−7^	7.28 ± 0.138	3.128×10^−7^

a: data obtained from 4 experiments, ED_50_ of ACh is expressed as molar, *p* < 0.05

+Et: in the presence of endothelial cells,

−Et: in the absence of endothelial cells

**Table 4:** Effect of inhibitors on vasorelaxant activity of *Spilanthes acmella* Murr. extracts.

Compound	Vasorelaxant activity							
	−Inhibitor		+l-NAME (1 mM)		+INDO (1 mM)		+l-NAME (1 mM) + INDO (1 mM)	
	R_max_ (%)	ED_50_ (mg/mL)	R_max_ (%)	ED_50_ (mg/mL)	R_max_(%)	ED_50_ (mg/mL)	R_max_ (%)	ED_50_ (mg/mL)
ACh^a^	121.74 ± 1.440	9.990×10^−7^	81.34 ± 0.770	5.455×10^−7^	68.78 ± 0.919	4.575×10^−7^	0	-
Hexane extract^a^	69.05 ± 0.693	5.016×10^−7^	31.29 ± 0.619	3.163×10^−7^	26.64 ± 0.768	3.162×10^−7^	0	-
Chloroform extract^a^	94.25 ± 0.873	5.278×10^−7^	51.62 ± 0.706	1.598×10^−6^	46.70 ± 0.511	6.661×10^−7^	0	-
Ethyl acetate extract^a^	85.84 ± 1.196	8.552×10^−8^	52.95 ± 0.976	7.804×10^−7^	44.48 ± 0.350	3.133×10^−7^	0	-
Methanol extract^b^	65.56 ± 0.535	9.444×10^−7^	33.24 ± 0.608	1.404×10^−6^	32.19 ± 0.420	1.072×10^−6^	0	-
SNP^b^	120.84 ± 1.176	3.164×10^−7^	116.70 ± 1.290	3.167×10^−7^	112.93 ± 0.613	3.155×10^−7^	104.98 ± 1.407	3.165×10^−7^

a: data obtained from 5 experiments,

b: data obtained from 6 experiments

ED_50_ of ACh and SNP are expressed as molar, *p* < 0.05, −Inhibitor: in the absence of l-NAME or INDO

**Table 5:** Radical scavenging activity and NBT inhibition of extracts.

Extract	% Radical scavenging activity^a^ (DPPH assay); 200 *μ*g/mL	% NBT inhibition^b^ (SOD assay); 200 *μ*g/mL
Hexane	4.90	0.41
Chloroform	29.82	57.92
Ethyl acetate	47.90	33.05
Methanol	47.76	47.02

a: *α*-Tocopherol was used as a positive control.

b: Native SOD (8.1 U) from bovine erythrocytes was used as a positive control.
